# Supplementary material for: Preparation and Characterization of PHBV/PCL-Diol Blend Films
Source: Polymers (Basel). 2023 Dec 13;15(24):4694. doi: 10.3390/polym15244694 (PMC10747782; doi:10.3390/polym15244694)
Supplement: Supplementary file 1 [file polymers-15-04694-s001.zip › polymers-2735908-supplementary.pdf]

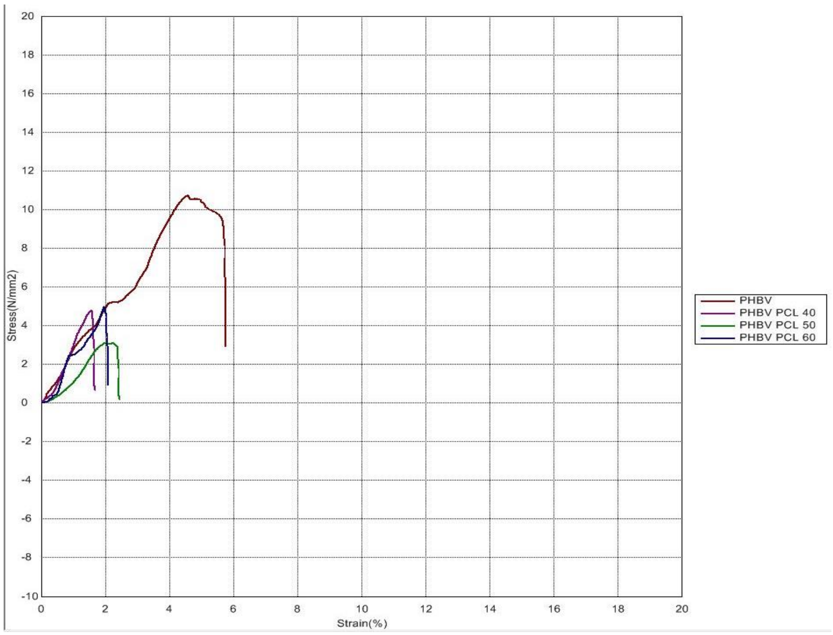

Figure S1. Tensile Stress-Strain diagram for neat PHBV; PHBV/PCL-diol 60%; PHBV/PCL-diol 50 % and PHBV/PCL-diol 40 %.
